# The neural processing of taste

**DOI:** 10.1186/1471-2202-8-S3-S5

**Published:** 2007-09-18

**Authors:** Christian H Lemon, Donald B Katz

**Affiliations:** 1 Department of Anatomy and Neurobiology, University of Tennessee Health Science Center, 855 Monroe Ave., Suite 515, Memphis, TN 38163, USA; 2 Department of Psychology and Volen National Center for Complex Systems, Brandeis University, Volen 208/MS 013, 415 South St., Waltham, MA 02454, USA

## Abstract

Although there have been many recent advances in the field of gustatory neurobiology, our knowledge of how the nervous system is organized to process information about taste is still far from complete. Many studies on this topic have focused on understanding how gustatory neural circuits are spatially organized to represent information about taste quality (e.g., "sweet", "salty", "bitter", etc.). Arguments pertaining to this issue have largely centered on whether taste is carried by dedicated neural channels or a pattern of activity across a neural population. But there is now mounting evidence that the timing of neural events may also importantly contribute to the representation of taste. In this review, we attempt to summarize recent findings in the field that pertain to these issues. Both space and time are variables likely related to the mechanism of the gustatory neural code: information about taste appears to reside in spatial and temporal patterns of activation in gustatory neurons. What is more, the organization of the taste network in the brain would suggest that the parameters of space and time extend to the neural processing of gustatory information on a much grander scale.

## Introduction

In general, there are two models of spatial coding that have been proposed to account for the neural representation of taste information. One viewpoint, known as "labeled-line" theory, proposes that neurons encode taste in a binary fashion: when cells are active (i.e., turned "on") they signal the presence of a particular stimulus feature, in this case a single taste quality [1,2]. When these same neurons are quiescent or "off", a stimulus that evokes this particular quality is absent. Thus, the activation of a cell serves one and only one purpose under the auspices of labeled-line theory. In contrast to this view, some have argued that taste is carried by a pattern of activity across a population of neurons [3,4]. In "across-neuron pattern" theory, individual neurons contribute to the representation of multiple stimulus qualities and quality information is signaled by the response of a neuronal population.

Although the coding debate has largely waffled between whether taste uses lines or patterns, traditional spatial coding models overlook information dependencies that could be present in the timing of action potentials or in time-dependent interactions among gustatory neurons. Yet the very nature of the organization of taste circuits in the central nervous system (CNS) as interactive networks arranged in series, in parallel and recurrently would impose temporal structure on the activities of neurons in any given taste nucleus or region. Such structure could serve various functions in the processing of taste, such as to evolve spatial representations about taste stimuli through time as related to various external and organismal variables. Here, we summarize recent developments that shed new light on how the parameters of space and time may contribute to the neural processing of taste information.

## Spatial processing: taste receptors and the brain

In some respects, a labeled-line mechanism is likely the least complex form of spatial coding that a sensory neural circuit could adopt. Interest in a line code as a plausible explanation of the operation of circuits for taste has been invigorated by the results of recent molecular and genomic studies of taste receptors. These investigations have identified two families of G-protein-coupled receptors, known as the T1r and T2r receptors, involved in the transduction of different taste stimuli. Members of the T1r class combine form heterodimeric, functional receptors that sense palatable taste stimuli. Specifically, the T1r3/T1r2 receptor recognizes some ligands described as sweet-tasting by humans whereas the T1r3/T1r1 receptor is involved in the detection of amino acid stimuli [5,6]. On the other hand, receptors of the T2r family are implicated for the detection of unpalatable, bitter-tasting ligands [7,8]. These receptors for sweet, umami and bitter stimuli have been found to be expressed in non-overlapping subsets of taste bud cells (TBCs) in oral epithelia, which has been interpreted as evidence of cellular specificity to a single stimulus quality [9-11]. Mice engineered to express receptors for a tasteless compound in TBCs that normally harbor T1r sweet or T2r bitter receptors display corresponding preference or aversion of this ligand [12,13]. Moreover, the expression of bitter receptors in T1r "sweet" TBCs results in behavioral attraction towards bitter ligands [12]. Some have argued that these findings indicate that individual TBCs respond to stimuli of only a single taste quality class and that information about a given quality is carried along one of a few dedicated, labeled neural channels [9,12-14].

Although the non-overlapping expression patterns of T1r and T2r receptors have been touted as evidence for labeled-line coding, other data paint a different picture of taste processing in the periphery. Functional studies using patch clamp electrophysiology and calcium imaging techniques have shown that many TBCs are broadly sensitive to stimuli of different taste qualities, with some TBCs responding to both sweet and bitter stimuli [15-17]. What is more, there is evidence for multiple receptors for sweet and umami stimuli [18,19], which tempers conclusions about the peripheral processing of these tastants drawn from studies of single kinds of receptors. Psychophysical studies have found no difference in detection thresholds for sucrose or monosodium glutamate between mice genetically engineered to lack the T1r3 receptor and wild-type controls [20], suggesting that T1r3-independent receptors are importantly involved in the detection of sweet and umami stimuli. Finally, there is now evidence suggesting that taste cells exchange information with neighboring cells within a bud and that there are separate populations of cells for sensing taste stimuli and communicating with afferent nerves [21-23]. This raises the possibility that information from taste receptor cells with different tuning properties could converge onto common cells in the taste bud for transmittal to the brain [23]. Processing within taste buds could potentially muddle the interpretation of receptor gene expression data as showing dedicated "lines" for taste qualities. Further studies of routes of communication within taste buds will shed light on the intricacies of interactions among TBCs.

**Figure 1:**
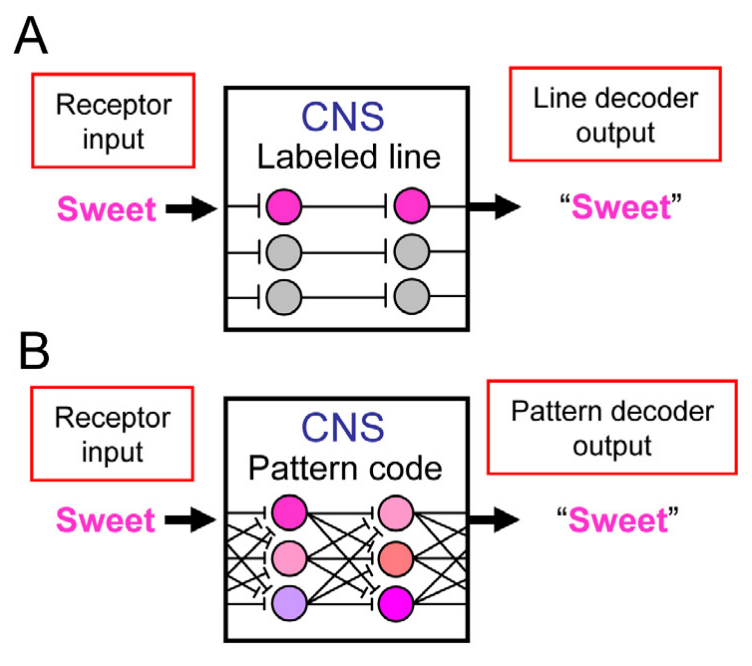
**There are multiple configurations of central taste circuits that could account for the perceptual consequences that follow stimulation of specific taste receptors.** In hypothetical model **A**, input from taste receptor cells that express sweet receptors is encoded along a labeled line in the central nervous system (CNS): information about a sweet stimulus is received exclusively by central neurons that respond only to sweets. A central labeled-line "decoder" could then know that a "sweet" stimulus is present when the sweet "line" is active. In model **B**, input from taste receptor cells that detect sweets is distributed across neurons and represented by a pattern code in the CNS. Here, a sweet stimulus produces a unique pattern of activation across cells. A central pattern decoder could recognize that a sweet stimulus is present through knowledge of this pattern. Under either coding strategy, the stimulation of sweet receptor cells results in the correct recognition of a sweet stimulus.

Taste receptors and TBCs are involved only in the earliest stages of gustatory processing and constitute a small fraction of the neural mass involved in the signaling and representation of taste information, which obviously and critically involves the activities of neurons and neural networks downstream in the central nervous system. Unraveling the logic of taste information processing will require understanding of how input from receptors is handled by circuits in the CNS, which cannot be deciphered from studies of taste receptors themselves. One can intuit that central networks for taste could be configured in any of a number of ways to "encode" input from receptors and give rise to an appropriate perceptual or behavioral output. Obviously, the brain must "know" the coding strategy employed by these circuits in order to "decode" the input and produce the appropriate response. But the organization of central networks for taste cannot be effectively elucidated by simply establishing their input (i.e., taste receptors) and output (i.e., behavior) relations. Thus, although the stimulation of TBCs that express sweet receptors, for example, will undoubtedly result in the transmission of a "sweet message" to the brain, the perception of sweetness would follow *regardless *of whether this signal is encoded along a labeled line, by a population code or a yet to be defined mechanism in the brain (Figure 1).

How are central neural circuits for taste organized to encode information about stimulus quality? Pursuing an answer to this question has been complicated by the pervasive *multisensitive *nature of central gustatory neurons. That is, numerous investigations have shown that central networks for taste are composed of categories of neurons that are generally broadly responsive to stimuli of different taste qualities. At odds with the line hypothesis drawn from studies of the expression patterns of T1r and T2r receptors, several studies have indicated that categories of central gustatory neurons that are strongly responsive to sweet or bitter stimuli are not specifically-tuned to only these kinds of tastants, displaying robust sensitivity to stimuli such as sodium salts (i.e., "salty") and acidic ("sour") solutions [24-27]. A neurophysiological study of how neurons in the nucleus of the solitary tract (NST) process bitter taste information conveyed by the VII^th ^nerve, which provides input critical for behavioral taste discriminations [28], found that the category of neuron that responded most strongly to bitter tastants, such as quinine, denatonium benzoate and papaverine, responded just as well to moderate concentrations of the sodium salts NaCl and NaNO_3 _[26]. From the perspective of a labeled-line code, that NaCl drives these bitter most-responsive neurons just as effectively as a many strongly bitter stimuli, such as quinine, would predict that NaCl should elicit a prominent "bitter" sensation that is common to quinine. However, rats do not behaviorally generalize between the tastes of NaCl and quinine in conditioning paradigms [29,30], suggesting that these stimuli are perceived by rodents as independent. In addition, rats prefer moderate concentrations of NaCl whereas they clearly avoid suprathreshold concentrations of quinine [31]. Yet if we attend solely to the output of bitter most-responsive neurons, which according to the line hypothesis should allow us to detect bitter tastes, we would not be able to tell whether NaCl or quinine was present on gustatory epithelia. A more recent study of the NST reported the presence of cells selective for bitter tastants [32]. But the majority of the bitter-sensitive neurons described in [32] were shown to receive taste input from the IX^th ^nerve, which is thought to contribute more to oromotor reflexes than to taste quality identification [28,33]. Clearly it becomes difficult to reconcile the neural representation of the qualitative identity of bitter tastants by considering only those neurons that respond most effectively to such stimuli.

That neuron categories or "types" are multisensitive suggests that the output of any single neuron class alone can only provide equivocal information about taste quality [34,35], which has implications for how central gustatory circuits could be organized to represent information about tastants. But before going further it is important to carefully consider what analyses of neuron *types *can actually tell us about neural information processing. It has been commonplace in gustatory neurophysiological studies to use as the unit of analysis the neuron type, which reflects the pooled response of neurons of a common category. These categories are typically defined by grouping cells on the basis of their best stimulus or through multivariate procedures that cluster neurons based on similarities among their response profiles to a set of stimuli. Analyses in which neuron type is a primary factor seemingly assume that it is the pooled response of a group of neurons that the brain would make due with in order to decipher stimulus input. But how would the brain pool the activities of neurons of a common type? Would the brain need to attend to all cells of the group or only a subset? Moreover, does the pooling scheme used by the brain adhere to experimenter-imposed categorizations of neurons? Or does the brain simply "readout" the activities of gustatory neurons on an *individual *basis? Of course, there are no clear answers to any of these questions. How the brain would pool the activities of gustatory neurons in the NST, for example, would likely be dependent on the specifics of synaptic connections between these cells and follower neurons in the parabrachial nucleus, a topic which is not well understood. Further, the average response of a neuron type could potentially under- or overestimate the tuning properties of individual cells. Thus, evaluating the coding performance of gustatory neurons is likely best indexed through understanding the information-handling limits of the *individual *cells themselves, which would also bear on the stimulus detection performance that could be achieved through pooling their activities in some way. Defining these limits requires knowledge of how reliably individual gustatory neurons respond to stimuli over time and across trials, a topic that has received only scant attention in the literature (but see [36]).

Just as important as within-neuron response variability, one must consider the length of time over which taste responses are measured. Many studies of taste processing have quantified the activities of gustatory neurons based on spike counts measured over 5 or 10 second stimulus-response windows. Yet it is important to acknowledge that this period is exceedingly long relative to amount of time that it takes the nervous system to arrive at a perceptual judgment about taste stimulus quality. Behavioral studies using conditioned avoidance procedures have shown that rats can recognize *and *respond to taste stimuli in less than 1 second following contact [37-39]. What this means is that necessary and sufficient information about stimulus quality is embedded in the spiking activities of gustatory neurons during the first few hundred milliseconds of evoked activity. This brief window containing critical information about stimulus identity may correspond to only a few or several action potentials maximum in many gustatory neurons when they are under taste drive.

**Figure 2:**
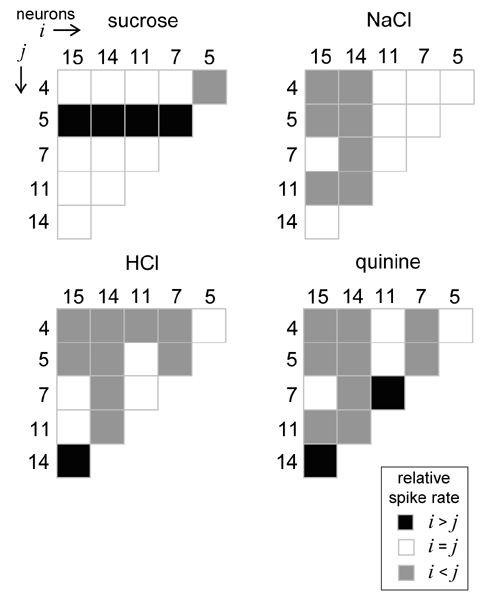
**Stimuli of different taste qualities produce unique patterns of relative firing among central gustatory neurons during the first second of stimulus processing.** Here, spiking rates to oral stimulation with sucrose (a prototypical "sweet" stimulus), NaCl ("salty"), HCl ("sour") or quinine ("bitter") were compared between taste neurons recorded from the rat NST using a theoretic technique based on statistical decision theory. This model bears on whether different cells fire at similar or reliably different spike rates when under the drive of a particular stimulus. The outcome of this analysis as applied to all possible neuron pairs among six randomly-selected cells is represented graphically as a set of half-matrices. A blackened matrix element represents that the *i*^th ^neuron (denoted along the matrix columns) of a particular pair fired at a detectably faster rate than the *j*^th ^(rows). A non-shaded element denotes similar spike rates (not different) between neurons, whereas halftone shading indicates that the *j*^th ^fired detectably faster than the *i*^th^. It can be seen that different stimuli produce unique relative response relationships among these cells. A downstream processor of these neurons with knowledge of the stimulus associated with each response relationship could, in principle, compute discriminations among these stimuli. Reprinted from [40], with permission of the *Journal of Neuroscience*.

Taking such issues under consideration, a recent neurophysiological study used a theoretic technique applied to individual rat NST neurons to explore how variability in the spiking rates of single cells during the first second of stimulus processing could impact the ability of spike rate to predict stimulus identify [40]. In all neurons tested it was found that stimuli of different taste qualities produced variable and overlapping distributions of spiking rates to the extent that the response of an individual neuron just after stimulus contact was an unreliable indicator of stimulus quality. This finding suggests that it could prove difficult, if not impossible, to decode stimulus input during this window by attaching taste messages to dedicated neurons or groups of them and simply reporting the message assigned to a processing unit when activated. Yet further analyses performed in [40] revealed that different tastants produced unique relative spiking relationships among several NST neurons compared in parallel. A "reader" that attends to this information and knows the stimulus associated with each pattern of relationships could, in principle, compute discriminations among different tastants (Figure 2). These data present a tenable model of how the gustatory system could use spatial coding to compute stimulus quality: rather than assigning meaning to individual neurons or categories of them, central gustatory circuits could signal quality information through the relative activities of multiple neurons in parallel. Other data have been argued to support this notion as well [4,34,35].

Although intriguing, the model presented in [40] (Figure 2) presents a description of how a spatial neural representation could *potentially *work in taste. The model shows that information about tastant identity could be extracted by a hypothetical reader that compares NST neurons under a theoretical framework. But it is, of course, unknown whether or not the nervous system would adopt a similar algorithm to register tastant identity. Understanding exactly how taste neurons are being "readout" by the brain will require knowledge of the architecture of networks linking these cells to downstream neurons and nuclei and the information transfer functions used in these circuits. The specifics here remain to be worked out.

## Time and interactive processing in taste

Historically, most models of gustatory coding have not attended to information that could be carried by dependencies on the timing of neural events. In fact, strict across-neuron pattern and labeled-line models assume that time matters very little in "gustatory coding." Evidence supporting this assumption is actually scanty, however. The use of 5–10 sec firing rate averages has, for the most part, been adopted by necessity rather than design, as taste neurons are often only held through single presentations of individual stimuli. While some studies report correlations between overall firing rates and taste-related behaviors among the members of large stimulus batteries, these correlations are moderate at best and describe only broad similarities between tastes [41]. Furthermore, the oft-cited fact that rodents can, under some circumstances, demonstrate recognition of a taste in ~200 msec [39,42] fails to serve as a strong indictment against temporal coding for at least 2 reasons: 1) this result causes equal problems for all current coding schemes – given that taste information arrives at the NST relatively slowly [43] and that taste responses are relatively low-firing-rate phenomena, neurons only have an opportunity to fire a few spikes in the first 200 msec that a taste is on the animal's tongue – a paucity of information for the purposes of reliable recognition of an activated neuron or spatial pattern; and 2) many taste-related behaviors occur only on a time-scale an order of magnitude higher than that described in the above-mentioned studies [44] – the code produced depends on the attentional state of the animal [45], along with many other task-specific variables.

There are several reasons to consider time in gustatory coding, meanwhile, above and beyond the fact that different taste behaviors require differing amounts of stimulus processing time. First, networks in the NST [46,47] and beyond, including larger networks of feed-forward and feedback connections [48-51], almost ensure that taste processing and coding will be modulated through time as neurons receive asynchronous input from multiple sources (this topic will be returned to shortly). In addition, it is likely that taste coding has a temporal aspect because most other sensory responses have been shown to do so [52-54].

What little evidence has been collected thus far suggests that gustatory neurons do respond to tastes with time-varying patterns of activity, at both the brainstem [36] and cortical [55,56] levels. To some extent this result is obtained because the collection of multiple trials of data, which is required for complex analysis, reveals subtle, phasic, and multi-phasic responses that are missed in overall rate analyses of single trial datasets. Such datasets also reveal that response profiles determined from experiments in which each taste was delivered only once or twice are frequently overly influenced by trial-to-trial variability in responsiveness [36], for which CNS neurons are notorious. Thus, reliance on large, tonic responses causes researchers to mischaracterize taste coding both in what is observed and in what is missed.

Adherents to spatial coding hypotheses deem the subtler, time-varying modulations observed in taste responses to be either "noise" or "unimportant." Such conclusions, however, are contradicted by at least two types of studies: those demonstrating that temporal codes carry specific, useful information, and those showing that animals can make taste judgments based solely on temporal codes.

As to the first of these types of studies, taste codes recorded from awake animals do not simply vary through time – they appear to "multiplex" information [55,57], as has also been shown for both visual [58] and olfactory responses [59]. That is, early portions of the taste responses, at least in cortex, convey information about taste quality, whereas later portions convey information about taste palatability. More recent data suggest that changes in these "late phase" responses are specifically related to changes in taste palatability, measured in terms of orofacial behaviors [45]. This serves as evidence that taste temporal coding may reflect the processing that the taste receives as the animal decides what it thinks of the taste.

Furthermore, examinations of ensemble responses reveal that what appears in single neuron records to be random trial-to-trial variability is in fact coherent at the population level. When spikes and firing rate changes in cortical neurons are related to spikes and changes in other, simultaneously recorded neurons (instead of to the onset of a stimulus), they can be seen to be progressing through taste-specific series of states that "evolve" at different rates on different trials [60]. The state sequence provides significantly better information about the taste delivered in a particular trial than do methods based on overall rates (or even time-varying PSTHs). This and the previous study suggest that the temporal codes are important to understanding the processing of tastes. While it is clear that gapes and licks can be produced by brainstem central pattern generator (CPG) circuits, lesion studies show that a large network of forebrain regions – prefrontal cortex, amygdala, and hypothalamus, at least – is responsible for making decisions about palatability [61-63], and thus for deciding which brainstem patterns are produced in the intact animal; as an analogy think of the control of walking, which can be done by spinalized mammals and yet intrinsically involves cerebellar and cortical control mechanisms [64].

But of course absolute proof requires experiments showing that temporal codes are used, not simply that they can be used. Two recent studies are exciting in this regard. In one, researchers electrically stimulated the NTS of rats, using a temporal pattern of spikes that had been previously recorded in response to a taste, as those rats drank water. The rats responded to the water as if it was bitter when a quinine pattern of stimulation was delivered [65]. Furthermore, a conditioned aversion to sucrose generalized to water consumed simultaneously with sucrose pattern delivery, but not to the delivery of other taste patterns. These data provide compelling evidence that rats use temporal codes for taste.

*Manduca *caterpillars also appear to make use of temporal information when identifying tastes [66]. These critters have a few transductive elements (sensillae) that respond to a wide array of bitter stimuli; these sensillae respond to aristolochic acid (AA) with an accelerating pattern of spikes, and to caffeine with a decelerating pattern of spikes. When efforts are made to equalize the overall spiking responses to these stimuli (i.e., the spatial codes), the caterpillars still distinguish between AA and salicin (another bitter taste that provokes a decelerating neural response) but not between caffeine and salicin. These data strongly support the value of time in taste.

The activity of neural networks provides a plausible mechanism for most of the temporal coding phenomena described above. Studies that have used multi-electrode recordings to reveal interactions between taste neurons [67-70], and those that have used a combination of stimulation/inactivation and recording to reveal feedback influences on NTS and PbN taste responses [48-51], demonstrate the reality of network processing in taste. Clearly, taste neurons "talk" to each other, and this conversation goes on between varieties of neurons within single brain regions, in convergences of neurons with disparate response patterns on single downstream targets, and in modulation of basic responses by forebrain neurons carrying more highly processed information. It is almost inevitable that neural interactions will cause modulation of taste responses through time.

There is evidence that the "code" for taste in the brain could involve both spatial [71,72] and temporal aspects of the activities of gustatory neurons. But the parameters of space and time are also critical to gustatory coding on a much larger scale. Taste processing is a network-level event, involving distributed CNS structures that engage one another in time-dependent fashion. It is this interactive processing between the nodes of the taste system that regulates information flow throughout the central gustatory neuraxis, ultimately shaping and evolving the neural "code" for taste relative various parameters of ongoing perceptual and behavioral processing. Understanding such neural interactions could provide a compelling window to the organization of circuits for taste, although our knowledge here is still in its infancy.

## Competing interests

The authors declare that they have no competing interests.

## Authors' contributions

C.H.L. and D.B.K. drafted the outline for this manuscript. C.H.L. wrote the Abstract, Introduction, the section entitled "Spatial processing: taste receptors and the brain", and the concluding paragraph. D.B.K. wrote the section entitled "Time and interactive processing in taste". C.H.L and D.B.K. edited the full manuscript.
